# Exploring the employment experiences of young adults with multiple minoritized identities: A qualitative study focusing on race and non-apparent disabilities

**DOI:** 10.1371/journal.pone.0313295

**Published:** 2024-11-01

**Authors:** Sally Lindsay, Natanela Dain, Shaelynn Hsu

**Affiliations:** 1 Bloorview Research Institute, Holland Bloorview Kids Rehabilitation Hospital, Toronto, Canada; 2 Department of Occupational Science & Occupational Therapy, University of Toronto, Toronto, Canada; 3 Rehabilitation Sciences Institute, University of Toronto, Toronto, Canada; University of Amsterdam, NETHERLANDS, KINGDOM OF THE

## Abstract

Youth with disabilities often encounter many challenges in securing employment. Although the common barriers that youth face are well documented, little is known about the experiences of youth who have multiple minoritized identities in finding and maintaining employment. Youth with disabilities who belong to racial minoritized groups may encounter ableism and racism and other forms of discrimination at work. Exploring the experiences of racial minoritized youth with non-apparent disabilities is important given the growing concern about social inequities that are linked to disadvantage and differential access to resources such as employment. We used a qualitative design involving semi-structured interviews with 19 youth (13 women, 3 non-binary, 3 men), aged 17–30 (mean 23.5 years). An inductive thematic analysis was applied to analyze the data. We identified the following themes: (1) challenges finding and maintaining employment; (2) the extent of safety and comfort with disclosing minoritized identities in the workplace (i.e., comfortable disclosing; uncomfortable disclosing; did not need to disclose and/or hiding identities); (3) workplace discrimination based on minoritized identities (i.e., ableism, racism, ableist racism, gendered ableism, racist and gendered ableism); (4) impact of discrimination (i.e., negative affective outcomes, social and work adjustment, impact on professional development) and coping strategies (i.e., advocacy, networking, peer support); and (5) advice for youth and employers. Our study highlights the extent of racism and ableism that racial minoritized youth with non-apparent disabilities experience in the workplace and the importance of rehabilitation clinicians supporting their inclusion.

## Introduction

Having a diverse workforce is essential for a prosperous global economy and employers are increasingly looking to enhance the diversity and inclusion of their staff [1, 2]. The social movement towards achieving enhanced equity, diversity and inclusion in the workplace is a reflection of the rising need to address barriers experienced by people from minoritized groups, such as people with disabilities and those who identify as racialized [3–5]. Intersectional approaches to understanding the experiences of minoritized groups are important because they tend to view disability and race as mutually reinforcing rather than as individually and separately [6–8]. For example, those who belong to multiple minoritized identities (e.g., disabled, racialized) often experience complex challenges and systemic factors that are not attributed to a single identity or to one form of discrimination or oppression [6, 9].

Research on intersectional identity and employment outcomes indicates how racial and ethnic minoritized individuals with disabilities often experience multiple forms of discrimination including racism and stereotyping at the individual and systemic levels, while also lacking access to educational and employment opportunities [10]. Such experiences of oppression and discrimination can negatively impact mental health, vocational development and career pathways [11, 12]. Racial minoritized people with disabilities often mask or hide their disability and often do not disclose to others out of fear of stigma, discrimination, or differential treatment [10–12].

Youth and young adults with disabilities commonly experience barriers in finding and maintaining employment and often have higher unemployment rates compared to youth without disabilities [3]. In Canada, where this study was conducted, youth with disabilities comprise approximately 14% of those who identify as belonging to a racial minoritized group [13]. Exploring the experiences of racial minoritized youth with disabilities is relevant given the growing concern about social inequities that are linked to disadvantage and differential access to resources such as education and employment [14, 15]. Research consistently highlights racial disparities in accessing employment programs, including a lower likelihood of receiving supports and vocational rehabilitation services compared to white youth [14, 16–18]. Racial minoritized youth are often in greater need of social services and supports but they commonly encounter many challenges in accessing services (e.g., stigma, discrimination, language barriers) [14, 19]. Recent research highlights that racial minoritized youth with disabilities often experience worse employment outcomes compared to white youth with disabilities [14]; they are less likely to attend post-secondary education and to secure meaningful employment. Such trends cause concern because a lack of supports after high school increases the risk of poverty and unemployment [14, 20]. Despite growing evidence of racial disparities and systemic barriers to employment for racial minoritized youth, relatively little is known about the lived experiences of racial minoritized youth with disabilities and especially their employment experiences [6, 14].

In this study we focus on non-apparent disabilities (also referred to as hidden or invisible because symptoms and impairments are not readily observable in most situations) [21]. Non-visible disabilities can include physical, mental and/or neurological conditions (e.g., autism, brain injury, epilepsy, anxiety and depression) that can result in difficulties with everyday functioning, activity limitations and/or participation restrictions that are not immediately obvious to others [22–24]. Some studies highlight the need for further research exploring how non-apparent disabilities, which can be concealed in certain contexts, may affect workplace experiences [22, 25, 26]. Unique challenges and considerations associated with non-apparent disabilities in the workplace that may affect decision-making around disclosure include stigma, discrimination, and lengthy processes for requesting accommodations [22, 27–29]. Such disclosure decisions could impact well-being, workplace accessibility and social inclusion [22].

Securing workplace accommodations often requires individuals to initiate the process by first disclosing their disability [27, 30, 31]. The fear of facing potential ableist reactions from co-workers could influence one to conceal their disability [27, 28, 32]. Disclosing a disability at work could be even more complex for racial minoritized youth who also encounter racism at the individual and systemic levels [14]. However, research exploring employment experiences and workplace disability disclosure among racial minoritized groups is lacking [22, 27]. Little is known about the potential additional and/or distinct barriers to obtaining employment and workplace inclusion that may exist for those with multiple minoritized identities who are living with non-apparent disabilities. It is important to consider how multiple social identities intersect to influence the employment experiences of individuals with non-apparent disabilities in the workplace, and how these may create further barriers to disclosure and receiving workplace accommodations [6, 10, 33–36]. In addition to those living with non-apparent disabilities [3, 37] racial minoritized youth with disabilities are particularly under-represented in employment research [6].

This study aimed to address this gap in understanding by focusing on the employment experiences of youth who have multiple minoritized identities including racial minoritized and non-apparent disabilities. Most previous research on the employment of youth with disabilities tends to view them as one similar group and/or focuses mostly on white youth samples [38]. Little attention has been paid to youth with disabilities who identify as belonging to a racial minoritized group. To date, research exploring race and employment among youth with disabilities has primarily been quantitative and focuses on employment rates and outcomes while less is known about the lived experiences in the workplace [14, 36, 39–42]. These important knowledge gaps are worthy of exploration because such minoritized populations may face unique challenges. By asking racially minoritized youth with non-apparent disabilities to share their lived experiences, our study aims to tie together several relatively unexplored areas of inquiry. This is a necessary first step for identifying potential targeted supports, practices and policies aimed at making labour markets and workplaces more equitable, fair, and inclusive for historically marginalized populations.

## Materials and methods

Our research question was: What are the employment experiences of racial minoritized youth with non-apparent disabilities? We conducted semi-structured qualitative interviews using an inductive thematic approach [43]. Institutional Research Ethics Board approval (REB) (#0164) and written e-consent was received from each participant prior to conducting the interviews.

### Sample and recruitment

A purposive sampling strategy was used to recruit youth who met the following criteria: (1) youth and young adults aged 15–30 [44]; (2) self-identify as living with a non-apparent disability (defined as impairments that are not evident or readily apparent to others, either because their manifestations are not visible or are not clearly linked to disability (e.g., psychological, learning or physical disability that can be hidden), or because they are episodic (such as epilepsy) [29, 30, 45]; (3) self-identify as belonging to a racial/ethnically minoritized group; and (4) have current or previous employment or job-seeking experience. This age range aligns with the common age at which youth start employment in the location where we recruited participants [46]. We chose this age range because we recognize that many youth and young adults may start their first jobs later than youth without disabilities and we wanted to capture a sample that also had sufficient work experience to draw upon [47, 48]. Our upper age range is also consistent with the extended time that young adults now spend in “emerging adulthood” [49].

Participants were recruited through flyers and invitation letters mailed to participants potentially meeting the eligibility criteria via our institutional research participant database. We also recruited through relevant disability and community organizations who agreed to share the study recruitment flyer. Individuals who reached out to the team expressing their interest in the study received an information letter via email. All participants were screened for eligibility capacity to consent (following the guidelines outlined by our Research Ethics Board) and given an opportunity to review and ask questions about the study through a brief Zoom or phone call, prior to receiving the e-consent form (using the REDCap software) to sign virtually, as required by our research ethics board. Participants received a $25 (Canadian) gift card as a token of appreciation.

### Data collection

Data collection took place from November 2022 to September 2023 and interviews lasted up to 73 minutes (average 52 minutes, standard deviation ± 15.45 minutes). Most participants were from a large urban area in Ontario Canada, where the majority of the population (~55%) identify as belonging to a racial/ethnic minoritized group [50]. All interviews were conducted remotely via Zoom with closed captions turned on. Participants had the option to leave their cameras on or off, in an effort to enhance the diversity and inclusion of participants [51]. We also had the interview questions on a shared screen and allowed some participants to type their answers if they chose to do so. Thirteen participants had their camera on during the interview, while five participants either chose to leave the camera off or had to turn it off during the interview. Two female researchers (one of whom belonged to a racial minority), with training in qualitative research and the topic area conducted the interviews. The interviewers had no conflicts of interest with the participants.

The interviews followed a semi-structured guide that was adapted from a previous study led by our team on workplace disclosure and accommodations [48], and questions were revised to reflect the present study’s focus on racialization, non-apparent disabilities, and intersectionality (see S1 File). Questions asked youth to share and reflect on how their identity impacted or might impact their job-seeking, employment, and workplace disclosure experiences, and asked participants to share advice and ideas for youth and employers regarding how workplaces might become more inclusive. The interview guide was co-developed with minoritized members of our research team (i.e., racialized, disabled) and a racial minoritized youth with a non-apparent disability. It was then piloted with additional youth with disabilities from multiple minoritized backgrounds for comprehensiveness and flow of questions prior to implementing it to all participants. Demographics (i.e., age, gender, disability type, race/ethnicity) were also collected (see Table 1). All interviews were audio recorded and transcribed verbatim using the Zoom transcription feature. Each audio file was verified by a researcher (by listening to the original audio file and checking the transcript) for accuracy before anonymization and subsequent data analysis.

**Table 1:** Overview of participant characteristics.

ID#	Age	Gender	Disability type (self-reported)†	Race / ethnicity (self-identified)	Education Employment status	Themes (subthemes)
1	30	Woman	Physical disability (hidden)*	Asian	• Graduate degree • Employed (temporary) full time	• Challenges with finding and sustaining employment • Extent of safety and comfort with disclosing (comfortable disclosing) • Workplace discrimination (racism) • Impact and coping • Advice for youth and employers
2	19	Woman	Autism	Indian (South Asian)	• Currently in university • Unemployed (previous work experience) • Currently volunteering	• Challenges with finding and sustaining employment • Extent of safety and comfort with disclosing (comfortable disclosing) • Workplace discrimination (ableism, racism, ableist racism, gendered ableism, racist gendered ableism) • Impact and coping • Advice for youth and employers
3	21	Woman	Autism (non-verbal) and mental illness	Asian / Chinese	• Currently in university • Employed part-time	• Extent of safety and comfort with disclosing (comfortable disclosing, uncomfortable disclosing minoritized identities, did not want or need to disclose) • Workplace discrimination (ableism, ableist racism, gendered ableism) • Impact and coping • Advice for youth and employers
4	25	Man	Autism	Black Canadian	• Bachelor’s degree • Employed full-time	• Extent of safety and comfort with disclosing (comfortable disclosing, uncomfortable disclosing minoritized identities, did not want or need to disclose) • Workplace discrimination (ableism, racism, ableist racism) • Impact and coping • Advice for youth and employers
5	25	Non-binary	Generalized anxiety disorder and persistent depressive disorder	East Asian Chinese	• College diploma • Employed casual/part- time (multiple positions)	• Challenges with finding and sustaining employment • Extent of safety and comfort with disclosing (comfortable disclosing, uncomfortable disclosing minoritized identities, did not want or need to disclose) • Workplace discrimination (ableism, racism, gendered ableism, racist gendered ableism) • Impact and coping • Advice for youth and employers
6	25	Woman	Juvenile arthritis (hidden)*	Jewish*	• Graduate student • Employed part-time	• Challenges with finding and sustaining employment • Extent of safety and comfort with disclosing (comfortable disclosing, did not want or need to disclose) • Workplace discrimination (ableism, racism, ableist racism) • Impact and coping • Advice for youth and employers
7	28	Woman	Cerebral palsy* learning disability, Attention deficit hyperactivity; sensory disability; brain injury	South Asian / Guyanese	• College degree • Employed (full-time)	• Challenges with finding and sustaining employment • Extent of safety and comfort with disclosing (comfortable disclosing, did not want or need to disclose) • Workplace discrimination (ableism, racism, ableist racism, racist gendered ableism) • Impact and coping • Advice for youth and employers
8	25	Woman	Mental illness and learning disability	East Asian Canadian	• Master’s student • Unemployed (unpaid student placement, previous work experience)	• Challenges with finding and sustaining employment • Extent of safety and comfort with disclosing (comfortable disclosing, did not want or need to disclose) • Workplace discrimination (ableism, ableist racism) • Impact and coping • Advice for youth and employers
9	22	non-binary	Mental illness (anxiety) and chronic illness (unspecified)	East Asian / Chinese	• Bachelor’s degree • Unemployed (previous work experience)	• Challenges with finding and sustaining employment • Extent of safety and comfort with disclosing (uncomfortable disclosing minoritized identities) • Workplace discrimination (Ableism, racism, racist gendered ableism) • Impact and coping • Advice for youth and employers
10	24	Queer / non-binary	Autism; chronic illness; mental illness (post-traumatic stress disorder)	East Asian Han Chinese	• High school diploma • Unemployed (previous work experience)	• Challenges with finding and sustaining employment. • Extent of safety and comfort with disclosing (uncomfortable disclosing minoritized identities) • Workplace discrimination (Ableism, racism, gendered ableism, racist gendered ableism) • Impact and coping • Advice for youth and employers
11	17	Woman	Attention deficit hyperactivity disorder; learning disability; social anxiety	West Indian / Afro-Caribbean (Black)	• Currently in high school • Employed (part-time)	• Challenges with finding and sustaining employment • Extent of safety and comfort with disclosing (comfortable disclosing, did not want or need to disclose) • Workplace discrimination (ableism) • Impact and coping • Advice for youth and employers
12	25	Woman	Bilateral hearing loss	East Asian / Chinese	• Bachelor’s degree • Employed (part-time)	• Challenges with finding and sustaining employment • Extent of safety and comfort with disclosing (comfortable disclosing, did not want or need to disclose) • Workplace discrimination (racism) • Impact and coping • Advice for youth and employers
13	17	Woman	Anxiety; Chromosome 13 deletion	Asian Chinese	• Currently in high school • Unemployed (previous work experience), • Volunteering	• Challenges with finding and sustaining employment • Extent of safety and comfort with disclosing (did not want or need to disclose) • Workplace discrimination (ableism) • Impact and coping • Advice for youth and employers
14	21	Woman	Mental Illness; learning disability; chronic illness	Middle Eastern / Jordanian	• Bachelor’s degree • Unemployed (previous work experience)	• Challenges with finding and sustaining employment • Extent of safety and comfort with disclosing (comfortable disclosing, uncomfortable disclosing minoritized identities, did not want or need to disclose) • Workplace discrimination (racism, ableist racism) • Impact and coping • Advice for youth and employers
15	25	Woman	Learning disability (ADHD), mental illness (depression), chronic illness (diabetes, asthma, leaky heart valve)	Chinese, East Asian	• Master’s degree • Employed (full-time and part-time positions), • Volunteering	• Extent of safety and comfort with disclosing (comfortable disclosing; uncomfortable disclosing minoritized identities) • Workplace discrimination (Ableism, racism, gendered ableism) • Impact and coping • Advice for youth and employers
16	27	Man	Physical Disability (wears prosthetic leg of right side, identified as being able to mask this)*	Bangladeshi, South Asian	• Bachelor’s degree • Employed (full-time)	• Challenges with finding and sustaining employment • Extent of safety and comfort with disclosing (uncomfortable disclosing minoritized identities) • Workplace discrimination (racism, ableism) • Impact and coping • Advice for youth and employers
17	22	Woman	Developmental disability	Filipino, South Asian	• Master’s degree • Employed (part-time)	• Challenges with finding and sustaining employment • Extent of safety and comfort disclosing (uncomfortable disclosing minoritized identities, forced to disclose) • Workplace discrimination (Racism, ableism, gendered discrimination, racist ableism, racist gendered ableism) • Impact and coping • Advice for youth and employers
18	21	Man	Developmental disability (autism)	Chinese and Jewish, Asian (mixed)	• High school diploma • Employed (full-time)	• Challenges with finding and sustaining employment • Extent of safety and comfort disclosing (comfortable disclosing minoritized identities) • Impact and coping • Advice for youth and employers
19	28	Woman	Autism spectrum disorder, social phobia, obsessive compulsive disorder, major depressive disorder	Black Afro-Caribbean, Grenadian	• Bachelor’s degree • Employed (full-time)	• Challenges with finding and sustaining employment • Extent of safety and comfort disclosing (comfortable disclosing minoritized identities) • Workplace discrimination (Racism, ableism, racist gendered ableism) • Impact and coping • Advice for youth and employers

* Note: identified that they keep their disability hidden

### Data analysis

We used an inductive thematic analysis approach to understand participants’ experiences and perspectives [43]. We followed the key steps of thematic analysis, beginning with data familiarization, which involved all authors reading through each transcript multiple times and taking notes on each transcript and initial reflections on key patterns and trends. The second and third authors were also involved in transcript verification, which is an important component of data familiarization [43, 52]. All authors contributed to the second and third steps involving an open, inductive coding of the transcripts and collating these codes around experiences of finding and maintaining employment and intersectional minoritized identities at work. The latter step occurred through meetings to discuss and compare initial codes and to identify a coding framework that could be applied within NVivo, version 12. The codes were reviewed by all authors and, with the research question guiding the analysis, an initial thematic framework responding to the research question was developed. We accomplished this during team meetings, in which patterns of codes and data items were identified and organized to formulate potential themes and sub-themes. For the fourth and fifth steps (i.e., reviewing, naming, and defining themes), two authors compared the thematic framework against the dataset in NVivo to identify any issues or need for reorganization. The team met again to review our notes and finalize the themes. We analysed relationships within and between the codes and themes. The second author applied the coding framework to all the transcripts in NVivo and the first author verified the accuracy of the application of the codes. Our team agreed that code saturation was achieved and that no new codes or meaning of the codes developed after extensive analysis [53].

We used various strategies to ensure trustworthiness and rigour [54, 55] including involving multiple coders and engaging in various peer-debriefing sessions. All authors reflected on their social positions, experiential viewpoints (e.g., child disability, sociology, health science, rehabilitation) and own potential biases during the data analysis. Some members of our team identify as belonging to a marginalized group, including one having a disability another a racial minority. We maintained detailed notes documenting key decisions and developments, including a journal with reflections following the interviews, transcript review, team meetings, and the data analysis process. Regarding dependability, we applied a consistent interview guide for all participants and kept notes for each interview. For confirmability, we frequently reviewed the transcripts throughout the analysis process to ensure the codes and themes reflected the participants’ experiences [56]. Additionally, we used exemplar quotes that reflected each theme and sub-theme while considering the whole context of each interview. We also followed the Consolidated Criteria for Reporting Qualitative Research guidelines (see S1 Checklist) [57].

## Results

### Participant characteristics

Our sample consisted of 19 youth and young adults, aged 17–30 (mean age 23.5), including 13 women, three non-binary and three men (see Table 1). Types of disabilities represented in our sample included autism (5), mental health condition (e.g., anxiety, depression, post-traumatic stress disorder, and other unspecified mental health conditions) (6), learning disability (5), physical disability (e.g., cerebral palsy, juvenile arthritis, prosthetic) (3), which they identified as non-apparent or hidden; attention-deficit/hyperactivity disorder (3), and hearing loss (1). Fifty-seven percent (11/19) of participants had multiple non-apparent disabilities. Regarding racial/ethnic identity, six identified as East Asian, four South Asian, three Black, one Jewish, one Middle Eastern, and one mixed race. Sixty-eight percent (13/19) of participants were currently employed while the remainder, 32% (6/19), had previous employment experience and were either looking for work and/or in school. Participants worked in various sectors including academia, healthcare, government, technology, media, manual labour, fast food, restaurants, retail, small family businesses and large corporations. Two youth were currently enrolled in high school, two had high school diplomas, two had a college diploma, two currently in university, six had a bachelor’s degree, five had a graduate degree or were currently pursuing one. Please note that we use the terminology that participants described to us along with their identity preferences. We recognize that there is much debate about using terms ‘disabled’ or ‘person with a disability’ but feel that it is up to individuals to decide how they wanted to be described, and it is important to respect their preferences.

### Overview of themes

We identified the following themes: (1) challenges finding and maintaining employment; (2) the extent of safety and comfort with disclosing minoritized identities in the workplace (i.e., comfortable disclosing; uncomfortable disclosing; did not need to disclose and/or hiding identities); (3) workplace discrimination based on minoritized identities (i.e., ableism, racism, ableist racism, gendered ableism, racist and gendered ableism); (4) impact of discrimination (i.e., negative affective outcomes, work adjustment, professional development) and coping strategies (i.e., advocacy, networking, peer support); and (5) advice for youth and employers. We recognize that the themes are inherently interconnected; however, they are discussed sequentially. Table 2 provides representative example quotes for each theme.

**Table 2:** Themes and example quotes.

Theme	Sub-theme	Representative examples
**1.** Challenges finding and maintaining employment	Challenges commuting	“The big considerations for me in finding employment is, I don’t drive. So, a lot of jobs already kind of removed because I can’t…get access to that. But also, it’s complicated by my anxiety affects me getting a license, but also it affects me accessing public transit.” (#5)
	Inaccessible job screening and interview process	“A part of me that was like, okay, what are you really looking for right now?. . . are you going to show this to a psychologist and be like she has indicators for a learning disability and throw my application out. (#7)
	Compatibility with job role	“You can get very stuck in a rut, because that is a desk job and doesn’t really suit me.” (#15)
**2.** Extent of safety and comfort with disclosing identities in the workplace	Comfortable disclosing	“If you’re in a positive space, you’re going to feel comfortable.” (#11) “If I perceive that it’s friendly and welcoming, I might apply. But if I find that it’s rigid and might have a lot of cliques, I may be less comfortable.” (#19)
	Uncomfortable disclosing	“I feel like whenever I have a job, I don’t tend to disclose my disability because it’s usually not something I feel is necessary to disclose unless it really comes up.” (#8)
	Did not need to disclose	“They (my co-workers) really don’t need to know.” (#13)
**3.** Workplace discrimination based on minoritized identities	Ableism	“I needed breaks because I just couldn’t keep being on the computer 24/7 and all of that…I tried to explain to her that it was a disability related break, and I need that to be able to perform effectively. But she had already made up her mind and was very upset and angry with me.” (#8)
	Racism	“When Black people come into the store, they’ll see me and my co-workers and if they’re Black they will talk to me first. Even yesterday, my co-worker is brown, and a brown man saw me and then her hijab is covering her shirt; So, then he saw me and looked at me, ignored me then looked at her and was like, can you help me? People are more comfortable with their own culture and their own race.” (#11)
	Ableist racism	“I would probably lean more on ensuring that my voice as a Jewish person was heard as opposed to my voice as a disabled person.” (#6)
	Gendered ableism	“ADHD is more common in boys, or so they say, but I think it’s because girls go undiagnosed…We struggle in our own ways, and then girls are generally better at masking things.” (#15)
	Racism and gendered ableism	“People like me we don’t get diagnosed because of the various culture and immigration and gender…when people do get diagnosed, they don’t have the factors that might lead them to be pressured into the high intensity work…You don’t get to see that representation often…We never have a confirmed role model.” (#2)
**4.** Impact of discrimination and coping	Negative affective outcomes	“I’m applying to jobs but…nobody wants me. Nobody wants to hire me…so I was like, I’m not good enough to get any kind of job and that really affected my mental health.” (#7)
	Work adjustment, barriers to diagnosis	“I’ve haven’t disclosed because…I wasn’t really sure yet at the time of my diagnosis…I made these mistakes (at work) and it led to a lot of frustration from my employers…I’ve almost gotten fired a few times…I did actually get fired once…Looking back, should I have said something?” (#17) “I feel that in the first place, me getting a diagnosis late, I think my race may have had to do with it…. So…being diagnosed late, I’m not sure where to start from, or where my real strengths are, which then influence my employment experiences.” (#19)
	Overqualified	“If you’re a disabled person who gets a job and then disclose your disability, you’re still at risk of being the first to get cut, if they need to lay off people…unless you are over-qualified and proving that…you make up for being disabled.” (#10)
	Advocacy	“It doesn’t matter if you have a learning disability; you can do it as long as you put your mind to it, and you just have to self-advocate.” (#11)
	Networking	“Networking is important, because you can see if the company, you’re working for is actually inclusive and accommodating.” (#9)
	Peer support and social bonding	“Seeking out people with a similar disability and seeing if there’s anything to glean from them.” (#12)
**5.** Advice for youth and employers	Compatibility with abilities	“Find if there’s a job that you think might be a right place—that is open to disclose in terms of…what you’re diagnosed with. Find something that you think is… suitable for your role” (#18)
	Representation in the workplace	“They can put people who are disabled even at the front offices who are working, and someone who is disabled works in maybe to look for a job and sees someone who is disabled working, they can feel encouraged” (#4)
	Education and training	“Having a panel of sort with people, living with disabilities would be really interesting and hearing people’s first-hand accounts of what it’s like living with that disability. Having a range of disabilities represented.” (#12)
	Onus on employer	“Initiate that conversation. Don’t put the onus on the disabled person.” (#10)

### Theme 1: Challenges finding and maintaining employment

Most racial minoritized youth with non-apparent disabilities experienced challenges, resulting from their minoritized identities (race and disability) at various stages of employment including looking for work and on the job. Such barriers occurred at the individual, family, and institutional levels. For example, youth explained how difficulties with public transit or driving limited their employment options. Youth expressed concerns about passing the job application screening criteria or getting through a job interview, which often caused them panic or anxiety attacks. Meanwhile, a challenge that some youth experienced on the job included the physicality of the work. For example, a youth with autism explained: “I felt so exhausted working once a week. Now that I have a diagnosis, and I know what it is, never again fast food…Maybe there’s types of work that I’m better suited to do and are more flexible in ways that are compatible with me” (#2).

Participants often had difficulty with finding employment aligning with their abilities. To illustrate, a youth said, “For people like us who are autistic, we really can’t do some tasks” (#4). Another participant with multiple disabilities explained their challenges with employment:

“Having so many disabilities at once will make you feel very discouraged to do anything, including school and employment…having ADHD, learning disability and anxiety is a lot because to work at a workplace you have to be a fast-thinker and you have to also socialize and so, you can’t be very isolated to yourself but people are not being friendly and that’s the challenge I’m having.” (#11)

### Theme 2: Extent of safety and comfort with disclosing minoritized identities

All youth described the extent to which they felt safe within their work environment and how this influenced their decision to disclose one or more of their minoritized identities (i.e., race and disability).

#### Comfortable disclosing

Participants explained why they felt comfortable disclosing (or potentially disclosing) one or more of their minoritized identities, mainly because they felt included within their workplace (i.e., inclusive employer/safe environment, having a similar identity to their co-workers and/or clients they served). Youth explained their decision to disclose their disability resulted from their employer focusing on clients with disabilities. For instance, one youth described: “We’re an organization that services people with disabilities. A lot of the staff are people that have lived experience with disability. So, [disclosing] was very easy to do” (#16). Another participant shared, “I’ll only disclose if I feel like they’re a group of people who have some experience with it (disability)” (#15). Other youth similarly expressed their comfort with disclosing their racial identity stemmed from having a similar racial minoritized identity to others within their workplace, which helped them to feel included. To illustrate, they said: “In terms of my ethnicity, most of the people I work with are also racialized…because (organization) serves East Asian and Asian communities…most people are similar to me” (#5). Participants appreciated working in a respectful environment and working with co-workers who had similar experiences. For instance, an East Asian woman explained: “If the employer values diversity, inclusion and equity; if they’re respectful then I think the environment would be easier for me as a person with a non-visible disability to disclose” (#8).

#### Uncomfortable disclosing minoritized identities

Participants described their discomfort with disclosing one or all their minoritized identities (i.e., disability, race and/or gender) in some work situations. Some youth told us how their discomfort was due to the stigma associated with their disability. For instance, a youth with multiple disabilities revealed, “The mental illness stuff I don’t mention at all” (#3). Meanwhile, a youth with autism explained they did not want to disclose their disability out of fear they would be seen as using their disability as an excuse to not do their work. A youth with multiple disabilities explained they did not want to disclose their ADHD at one of their workplaces “because I’m afraid of them thinking that I’m a less capable worker” (#15). Another youth with a physical disability expressed: “I do always want to let my employer know as best as they can what my conditions are, but I will say it feels weird many times for me to bring it up, because it feels like I’m not sure what the impact of that is going to be” (#16). Additionally, a non-binary East Asian youth felt vulnerable about disclosing their gender identity to others. Specifically, they shared, “I feel conflicted about telling them (co-workers) because I don’t know of the status of my safety if they would treat me differently” (#5).

Some participants were uncomfortable with disclosing but felt they had no choice to make their own disclosure decision (i.e., forced disclosure) when their disability identity became more of an issue. A youth explained, “I have social anxiety; I’m not a very social person. They (co-workers) felt offended by me not talking to them. I felt I was forced to tell them; look I’m not being rude; I’m just not a social person” (#11). Another youth with multiple disabilities needed to disclose their disability to their employer when they started to get sick and had lots of hospital appointments they needed to attend.

#### Did not want or need to disclose: Hiding minoritized identities

Youth did not feel the need to disclose some of all their minoritized identities, especially in certain work situations. For example, a youth with autism shared: “If hiding the fact that I am autistic will make me have a job, why not?*”* (#4). Another participant similarly explained, “The benefit of having it (disability) be invisible is the luxury to hide it when I want to…My ethnic identity is also, in the similar sense to my disability, very hidden” (#6). This youth also mentioned how her ability to work from home helped with not needing to disclose her condition because she could organize her own disability-related workplace accommodations. Another participant with multiple disabilities shared, “If you don’t have a serious disability, or something that’s not visible, it’s easier to get away with because you learn how to cope with it without them knowing” (#9).

Some youth tried to hide their disability identity in the workplace because they worried their parents would find out, and their condition was viewed with stigma within their family and/or culture. A participant shared: “My mom doesn’t want me to disclose my disability because she feels like there’s a lot of stigma attached to mental illness and she doesn’t want that to negatively impact my peer or employer interactions” (#8). Further, a Middle Eastern woman with multiple disabilities explained:

“From a cultural standpoint no one really talks about disability…sick people are people who did something wrong…It started at home then quickly transferred into other parts of my life, particularly my employment. My illness and disability are something I carried a lot of shame around…but my ethnic identity added an additional layer onto it” (#14).

Several youths explained how their parents were often in denial about their disability identity, which influenced their willingness to disclose their disability in the workplace.

### Theme 3: Workplace discrimination based on minoritized identities

Most participants experienced, or expressed concern about experiencing, workplace discrimination based on their minoritized identities (i.e., race and disability) including ableism, racism, ableist racism, gendered ableism, as well as racist and gendered ableism.

#### Ableism

Most racial minoritized participants experienced ableism (both at the systemic and individual levels) within their workplace or when trying to find work. Several participants spoke about the systemic ableist barriers they experienced. For example, a youth explained how “disabled people…we don’t get the same kind of opportunity” (#17). Many youths shared experiences of ableism at the individual level. For instance, an Asian woman with autism said, “I’ve been on teams before where people didn’t know how to talk to me, and they’d rather talk about me than to me” (#3). Another woman described how her identity influenced her everyday experiences at work because she would “get stares sometimes” (#13). A Black woman similarly shared, “I have a learning disability, and they try to belittle me and try to make me seem like I don’t understand stuff*”* (#11). Further, a South Asian woman with multiple disabilities revealed her experiences of ableism during the job interview process: “Standing there in front of them with four disabilities, and they were like, this is too much work. We don’t know how to accommodate you. That becomes way too difficult in the workplace as compared to school” (#7). Some youth explained the ableism they encountered probably resulted from the non-apparent nature of their disability. They pointed out that their employers and co-workers often perceived non-apparent conditions as invalid.

#### Racism

Most participants experienced racism within the workplace (from employers, co-workers and/or customers) and/or in looking for work. Multiple youths shared that they experienced systemic barriers and limited opportunities for employment due to their racial and ethnic identity. For instance, some youth felt judged based on their name at the job application stage. To illustrate, an East Asian non-binary youth described, “People who have Caucasian names are more likely to go past the resume process because of how they filter in the system” (#9). Another youth explained that coming from an immigrant family, they did not have equal access to network and opportunities in university and when entering the workforce. They shared: “From the first day I started university in that program, I was kind of behind, because that concept of who you know, I was already a few steps back…That makes it harder to have the same types of opportunities once you’re finished university” (#16).

Some participants reported experiencing racism in the workplace. A Guyanese woman shared: “It’s those kinds of cultural things where people are, like, you have an ethnic name and you’re Brown and you have curly hair…let’s take someone else. But that’s also something that is difficult because you can’t change that” (#7). Another youth similarly shared, “I’ve gotten comments about my race and sometimes they’re out of ignorance*”* (#12). Some youth drew attention to anti-Asian racism, during the pandemic which created more anxiety for them. They explained that during and after the pandemic:

“I feel very disconnected from my racial and ethnic identity. Partially that comes from racism, a basis of being denied it, being othered from it, feeling like it’s not something that I should be proud of, or be connected with…in terms of employment experiences I find it hard to notice subtle discrimination or prejudice or racism is happening to me because I’m already anxious about how others perceive me.” (#5)

#### Ableist racism

Some participants described how their disability intersected with their racial identity, and especially how this often led to ableist and racist experiences. Youth reported that racist ableism happened in various stages of their employment. At the job application stage, a youth shared: “I’m actually a racialized person and a woman with a disability. I need accommodations… they’re like, that’s not what we’re looking for” (#7).

Several youths described feeling as though belonging to multiple minoritized groups gave employers several reasons to discriminate against them. Participants shared how they felt like they “checked off too many boxes” (#10) because of their multiple minoritized identities. Some youth also differed in the ways they perceived their identity and how it affected their experiences in the workforce. Some participants revealed they had greater concerns about racial and ethnic discrimination than ableism. For instance, a Black autistic man said, “The fact that you are autistic, and you are Black I did find that the hardest thing…I may face some racial discrimination…Racial discrimination is something I think may really affect the chances of me getting employed” (#4). Another youth explained, “The fact that I’m from an ethnic minority or I’m visibly seen as though I’m an ethnic minority would largely influence their (employer’s) response into a negative stance…one extra thing for them to discriminate against me” (#14). Other youth felt their disability played a larger role in their experiences of discrimination compared to their race.

#### Gendered ableism

Participants described experiencing gendered ableism, which refers to discrimination based on the intersection of disability and gender identities. For example, an Asian woman with autism experienced microaggressions from their co-workers who felt she was hired based on her gender. To illustrate, they said: “When I first got into this program, I had people tell me I was a token hire. So that sits at the back of my mind a lot” (#3).

Some participants identified as a gender minoritized person and described how their gender identity intersected with their disability identity. They often had to consider the extent of their safety and social inclusion within their work environment. For example, an East Asian non-binary participant explained,

“I’m part of the queer community and I’m non-binary…disability and all aspects of my identity really affect how I move through the world, especially employment…For disability, sexuality, and gender it’s a question of disclosure. It’s a question of who is safe to tell this to…more traditional employment definitely feels more intimidating.” (#5)

Another non-binary youth with multiple disabilities, shared a similar experience: “As a queer disabled person, I need to take into account what is within my ability to do in the context of the job…whether or not this workplace is going to have a culture that is going to be welcoming towards me…a lot of people working in these sorts of professions aren’t very welcoming when it comes to a queer person in their workplace” (#10). Some other non-binary youth commented on the lack of representation of their sex/gender identity within the workplace. They shared, “I’d rarely see queer people. I have to go to especially queer spaces to find opportunities but there’s not many of those right now” (#9).

#### Racist, gendered ableism

Some youth mentioned how their minoritized identities (based on their race, gender, and/or disability) intersected in the workplace or in looking for a job. For example, a South Asian woman with multiple disabilities shared, “I’ve been discriminated against based on my colour. I’ve been discriminated against based on my gender, and based on my disability” (#7). Another participant explained their experience: “It’s already challenging with having to deal with disabilities and then there’s potential unconscious bias for being East Asian and/or queer…Most teams are headed by a white cisman…I don’t know a lot of diversity of intersectionalities…I feel it wasn’t diverse…no one was open about disabilities” (#9). Another youth described their experiences of their minoritized identities in the workplace: “It’s more of the intersection of being queer and disabled and racialized….It’s one of those things where you can either only be queer, you can only be disabled, or you can only be racialized. You can’t be all 3….If you are that’s because it’s something that you can technically hide” (#10).

### Theme 4: Impact of discrimination

All participants described the impact of workplace discrimination including negative affective outcomes, social and work adjustment, and impact on professional development. For example, youth reported experiencing negative affective outcomes, including feelings of frustration, hopelessness, and discouragement as they sought work. For instance, a participant noted that they “lost hope” because of the discrimination they face (#4). Another youth shared how systemic barriers to employment affected their sense of self-worth: “I was supposed to be job searching, but it was really difficult for me. It was so hard. I was deep in the depression. I could barely manage applying to one job per month, because it was so disheartening” (#5). Youth discussed social-related work adjustments, with six drawing attention to feeling exclusion, discrimination and unwelcome. For example, a participant shared how she felt after an employer reacted negatively to her disclosing and requesting accommodations. To illustrate, she felt: “pretty shitty…it just made me feel like it was my fault, like, I’m not getting the support I needed…I just feel very invalidated” (#8).

Additionally, some youth lacked opportunities for professional development, which they perceived resulted from ableism, racism, and/or gendered discrimination. Some youth even quit their jobs because of the discrimination they experienced, while another youth mentioned that after disclosing his disability and requesting accommodations, the company he worked for let him go. One youth similarly reported that after requesting accommodations, her employer gradually assigned her fewer tasks. Meanwhile, participants felt they needed to be overqualified to help overcome the multiple forms of discrimination they experienced. One youth shared their experiences attending large corporate events: “it’s mostly people that don’t look like me…it’s not a lot of diversity…it’s more difficult to stand out in that way…it always just feels like I have to work harder than everyone” (#9). Some participants explained how their late diagnosis negatively impacted their employment experiences, eligibility for employment training programs and/or access to employment resources.

#### Coping strategies

Participants described some coping approaches to dealing with racism, ableism, and other forms of discrimination in the workplace. Strategies included advocacy, networking, social bonding, familial and peer support. Participants highlighted the importance of focusing on their strengths and advocating for their achievements. A youth shared, “it’s really amazing what people can do in their circumstances, no matter how difficult they are, or no matter how many things are barriers for them” (#5). Most participants recommended networking and connecting with support groups as a coping strategy. They suggested that youth with disabilities “go to groups…like fairs and stuff for jobs” (#13) while leveraging their support network. A youth said, “talk with people…and use the resources you have available” (#8). Five participants noted the helpfulness of connecting with disability-specific groups. Meanwhile, youth spoke about the positive impact of bonding with others over their shared identities. For instance, a participant noted: “when I reached out to (co-worker with similar identity), it was actually nice, because…this is someone similar to me successful in something I want to pursue” (#2).

### Theme 5: Advice for youth and employers

All participants provided advice for youth and employers regarding finding employment. Youth recommended looking for work compatible with your disability, networking and finding relevant supports and/or a mentor to help with finding a job or requesting workplace accommodations. For example, a youth with multiple disabilities explained: “I want to see myself reflected in an organization…people who look like me…who share similar experiences” (#14).

Regarding advice for employers, youth recommended the need for further education and training on what disability means, how to accommodate various disabilities and how to create an inclusive environment that welcomes minoritized identities within the workplace. They also advocated for having automatic accommodations and opt out options rather than having to individually disclose and go through a lengthy and complex process of requesting accommodations. Youth recommended that employers be more transparent about workplace accommodations processes. Others felt the responsibility should not be on individual employees to change the whole work culture to be inclusive. For example, a youth explained: “don’t make diversity and inclusion its own thing. You just integrate it into the work culture and drive change. If you make it a chore, nobody is going to want to do it” (#10). Youth recognized the importance for employers not to assume (dis)ability status. For instance, a youth shared: “A lot of people think that disability or being part of minorities is exclusive to the individual itself and having them to navigate it…It’s not how you address the problem” (#14). Additionally, several youths wanted to see more diversity in the workplace, involving multiple minoritized identities.

## Discussion

This study focused on the employment experiences among racial minoritized youth and young adults with non-apparent disabilities. Exploring their perspectives is salient because racial minoritized youth with disabilities arguably encounter more challenges with securing and maintaining and navigating employment than white youth with disabilities [14, 38]. Our results showed that racial minoritized youth with non-apparent disabilities experience challenges with securing employment at the individual and systemic levels. The findings show consistency with recent research on employment barriers that youth with physical disabilities, autism, and other various types of disabilities experience [58, 59]. Previous quantitative research demonstrates that racial minoritized youth with disabilities often have poorer employment outcomes compared to white youth with disabilities [14, 36, 39–42]. Building on past quantitative studies of racial minoritized youth with disabilities, we implemented a qualitative study design and used semi-structured interviews to explore the unique perspectives and lived experiences of racial minoritized youth with disabilities. The youth in our sample described how belonging to multiple minoritized identities often intersected and created challenges for finding employment, disclosing, or hiding their disability and/or asking for workplace accommodations.

Many youths expressed the extent of safety and comfort with disclosing minoritized identities. Some youth experienced work environments where they felt unsafe, unwelcome, or discriminated against by co-workers and employers, which led youth to feel uncomfortable with disclosing one or more of their minoritized identities. Previous research (mainly with white samples) shows that workplace environment, particularly the extent of inclusivity, and perceived peer support from co-workers are important predictors of disclosure decisions [22]. Our findings align with other research focusing on workplace disability disclosure among adults with non-apparent disabilities, which demonstrate that non-apparent stigma and discrimination may influence disclosure and contribute to people concealing or hiding their minoritized identities [22]. Similarly, our results support previous research focusing on youth with physical disabilities, which discovered that comfort level with disclosing can be influenced by type of disability (i.e., visible/hidden), job type and industry, inclusive work environment, fear of stigma and discrimination and not disclosing on your own terms (i.e., forced disclosure) [3, 22, 59]. It is important to note that although our findings support existing literature, much of the previous literature was limited to predominantly white samples and those with visible disabilities.

Hiding non-apparent disabilities due to family/cultural-related stigma and shame among the youth in our study was consistent with other research on ableism among Asian youth with disabilities [60]. Experiences of cultural-related stigma and denial of disability could create additional barriers for these youth as they seek employment and could lead some racial minoritized youth with disabilities to feel particularly vulnerable with disclosing and advocating for their needs as they are relatively new to the workforce. Our findings suggest that given these unique barriers, racially minoritized youth with non-apparent may need additional supports (i.e., transitional programs, health coverage, transparent workplace accommodation processes) [3].

Our results showed that racial minoritized youth with disabilities experienced multiple forms of workplace discrimination including ableism, racism, ableist racism, gendered ableism, and racist/gendered ableism. Our findings align with research focusing on ableism and racism in employment among adults, which shows that people from racial minoritized groups with disabilities often encounter stigma, stereotypes or discrimination specific to their marginalized identities [6]. Such behaviours are often result from employers’ and co-workers’ assumptions about racially minoritized workers that affect their perceptions of disability and the need for workplace accommodations [6]. In particular, youth in our study highlighted how their employers or co-workers often questioned the legitimacy of non-apparent disabilities, which is consistent with the literature on racially minoritized adults with disabilities [6]. Our study revealed that racially minoritized youth with non-apparent disabilities often experience multiple forms of stereotypes and discrimination based on the intersection of their racial identities and their disability. These experiences of racist ableism at work occur both at the individual and systemic levels, show consistency with other research on racially minoritized adults with disabilities in the workplace [6, 61]. Moreover, our results align with recent quantitative studies on intersectionality that highlight how racial minoritized people with disabilities perceive significantly higher rates of discrimination, workplace harassment and earn significantly less than non-racial minoritized people without disabilities [36, 39–42, 61]. Although there was notably less attention given in our sample to gendered ableism, some participants reported experiencing systemic and individual-level discrimination based on the intersection of their racial and ethnic identity, their gender identity, and their disability, citing limited access to employment resources, differential treatment from their healthcare providers, and workplace harassment. Other research on youth with various types of disabilities highlights that gender can affect workplace ableism [3]. Despite labor laws aiming to address or prohibit ableism, most racial minoritized youth with disabilities in our sample experienced disability-related discrimination and/or racism in the workplace. This trend is concerning because research shows that ableism often results from stereotypes and negative attitudes, which can lead to prejudice and discrimination and a lack of support for people with disabilities who are left feeling marginalized [3].

Although we did not ask about it specifically, youth in our study described the impact that various forms of workplace discrimination had on them, including negative affective outcomes, poor work and social adjustment, and limited opportunities for professional development. These findings are similar to other recent research focusing on youth with various types of disabilities in experiencing a lack of job supports, social exclusion, and job turnover, underemployment and unemployment [3]. Our results contrast other research on workplace ableism among youth with various types of disabilities [3] in that there was no mention of pay discrimination or formal discrimination allegations. These findings suggest that there is an urgent need to understand why some young people who experience discrimination might not be making formal complaints. Additionally, there is a demand for more effective solutions that address workplace inclusion of racial minoritized people with disabilities in the workforce. Greater efforts are needed to address the systemic discrimination of people with disabilities in the workplace, including more accessible interviewing, screening, and hiring practices.

Our results shed light on how some racial minoritized youth implemented coping strategies to deal with ableism and racism in the workplace including advocacy, networking, and seeking peer support. Our findings somewhat align with the coping strategies that youth with physical and other various types of disabilities used to address challenges to finding employment such as job preparation, knowledge of supports and self-advocacy [58, 59]. It is important to highlight that youth with disabilities who belong to racial minoritized groups may experience more challenges with accessing resources and supports than with disabilities [14, 38]; however, additional research is needed to explore this further.

### Implications and recommendations

Our findings have several important implications for employers and service providers who support youth with disabilities. First, at a socio-cultural and systems level there is an urgent need for further advocacy of the inclusion of people with minoritized identities in employment. Decision-makers and employers should ensure that their policies regarding equity, diversity and inclusion in the workplace adequately address the needs of people with multiple minoritized identities. This involves actively removing barriers and examining ways in which they may be contributing to multiple forms of discrimination (ableism and racism) within their workplaces.

Second, there is a critical need for more inclusive and accessible job hiring process including screening and interviewing. For example, some youth expressed concern that their application would be automatically screened out if they did not have a white or Western name. The use of artificial intelligence in job applicant screening should consider and address possible bias and discrimination towards applicants with minoritized identities. Employers should also consider having a more flexible and easy-to-navigate workplace accommodation process so that the responsibility of disclosing a disability is not placed on individuals. It would also be helpful if employers offered more in-depth training regarding disability including lived experiences to create a more inclusive environment. Third, clinicians and service providers who support youth with finding employment could benefit from further training in intersectional identities. They can also help to connect youth to relevant employment resources and supports and support them with addressing any individual barriers they encounter in finding a job.

### Limitations and future research

Our findings should be interpreted with caution while considering some of the study’s limitations. First, our sample had an uneven gender and racial distribution, which could impact youth’s employment experiences. Additionally, the generalizability of the findings is limited given that we did not use conventional categories for race/ethnicity but rather allowed participants to self-identify. Further research should consider exploring men’s experiences and Black youth with disabilities in greater depth. Future research could consider exploring the experiences of ableism and racism within specific job types or industries. Additionally, the types of non-apparent disabilities in our sample varied and could impact youth’s employment experiences. It is also important to acknowledge that those with hidden disabilities may also be more likely to engage in hiding their condition, which could affect their experience of employment. Future research could explore the components of what makes a work environment inclusive to the extent that people with minoritized identities feel comfortable disclosing. Finally, more focus on short and longer-term outcomes of ableism and racism in the workplace and effective solutions for addressing and reducing discrimination.

## Conclusions

Our study explored the employment experiences of racial minoritized youth and young adults with non-apparent disabilities. Our findings highlighted the challenges that youth encountered with securing and maintaining employment. Youth described the extent of safety and comfort with disclosing minoritized identities in the workplace. Many youths felt the need to hide one or more of their minoritized identities. Most youth experienced some form of workplace discrimination based on their minoritized identities (i.e., ableism; racism; ableist racism; gendered ableism; racist and gendered ableism). Youth explained the impact of the discrimination they experienced including negative affective outcomes, social and work adjustment, and professional development. Some youth had coping strategies for dealing with stigma and discrimination such as advocacy, networking, peer support. Our findings highlight the extent of racism and ableism that racial minoritized youth with non-visible disabilities experience in the workplace and the urgent need for more effective strategies to enhance inclusion and reduce discrimination.

## Supporting information

S1 Checklist Coreq checklist. (DOCX)

S1 File Interview guide. (DOCX)
