# Supplementary material for: Exploring the employment experiences of young adults with multiple minoritized identities: A qualitative study focusing on race and non-apparent disabilities
Source: PLoS One. 2024 Nov 1;19(11):e0313295. doi: 10.1371/journal.pone.0313295 (PMC11530060; doi:10.1371/journal.pone.0313295)
Supplement: S1 Checklist — (DOCX) [file pone.0313295.s001.docx]

Supplemental Table 1

Consolidated Criteria for Reporting Qualitative Research (COREQ checklist)

| **No. Item** | **Guide questions/description** | **Location in manuscript** |
| --- | --- | --- |
| **DOMAIN 1: RESEARCH TEAM AND REFLEXIVITY** | | |
| *Personal Characteristics* |  |  |
| 1. Interviewer/facilitator | Which author/s conducted the interview or focus group? | Methods, para **5** |
| 2. Credentials | What were the researcher’s credentials? | Author list |
| 3. Occupation | What was their occupation at the time of the study? | Methods, para **4** |
| 4. Gender | Was the researcher male or female? | Methods, para **4** |
| 5. Experience and training | What experience or training did the researcher have? | Methods, para **4** |
| *Relationship with participants* |  |  |
| 6. Relationship established | Was a relationship established prior to study commencement? | Methods, para **4** |
| 7. Participant knowledge of the interviewer | What did the participants know about the researcher? | Methods, para **4** |
| 8. Interviewer characteristics | What characteristics were reported about the interviewer/facilitator? | Supplemental file 1 (interview guide) |
| **DOMAIN 2: STUDY DESIGN** |  |  |
| *Theoretical framework* |  |  |
| 9. Methodological orientation and Theory | What methodological orientation was stated to underpin the study? | Methods, para **1, 6** |
| *Participant selection* |  |  |
| 10. Sampling | How were participants selected? | Methods, para **2** |
| 11. Method of approach | How were participants approached? | Methods, para **2;** |
| 12. Sample size | How many participants were in the study? | Results, para **1** |
| 13. Nonparticipation | How many people refused to participate or dropped out? Reasons? | Methods, para **1** |
| *Setting* |  |  |
| 14. Setting of data collection | Where was the data collected? | Methods, para **4** |
| 15. Presence of non-participants | Was anyone else present besides the participants and researchers? | Methods, para **4** |
| 16. Description of sample | What are the important characteristics of the sample? | Results, para **1**; Table 1 |
| *Data collection* |  |  |
| 17. Interview guide | Were questions, prompts, guides provided by the authors? Was it pilot tested? | Methods, para **5** Supplemental File 1(Interview guide) |
| 18. Repeat interviews | Were repeat interviews carried out? If yes, how many? | N/A (no repeat interviews) |
| 19. Audio/visual recording | Did the research use audio or visual recording to collect the data? | Methods, para **4** |
| 20. Field notes | Were ﬁeld notes made during and/or after the interview or focus group? | Methods, paras **5** |
| 21. Duration | What was the duration of the interviews or focus group? | Methods, para **4** |
| 22. Data saturation | Was data saturation discussed? | Yes |
| 23. Transcripts returned | Were transcripts returned to participants for comment and/or correction? | No |
| **DOMAIN 3: ANALYSIS AND FINDINGS** | |  |
| *Data analysis* |  |  |
| 24. Number of data coders | How many data coders coded the data? | Methods, para **6** |
| 25. Description of the coding tree | Did authors provide a description of the coding tree? | Methods, para **6** |
| 26. Derivation of themes | Were themes identiﬁed in advance or derived from the data? | Methods, para **6** |
| 27. Software | What software, if applicable, was used to manage the data? | Methods, para **6** |
| 28. Participant checking | Did participants provide feedback on the ﬁndings? | No |
| *Reporting* |  |  |
| 29. Quotations presented | Were participant quotations presented to illustrate the themes/ﬁndings? Was each quotation identiﬁed? | Results (throughout); Table 2 |
| 30. Data and ﬁndings consistent | Was there consistency between the data presented and the ﬁndings? | Yes |
| 31. Clarity of major themes | Were major themes clearly presented in the ﬁndings? | Results (throughout); Table 2 |
| 32. Clarity of minor themes | Is there a description of diverse cases or discussion of minor themes? | Results (throughout); Discussion (throughout) |
