# Supplementary material for: Exploring the employment experiences of young adults with multiple minoritized identities: A qualitative study focusing on race and non-apparent disabilities
Source: PLoS One. 2024 Nov 1;19(11):e0313295. doi: 10.1371/journal.pone.0313295 (PMC11530060; doi:10.1371/journal.pone.0313295)
Supplement: S1 File — (DOCX) [file pone.0313295.s002.docx]

**S1. Interview guide**

**Interview guide (youth)**

Thank you for taking part in this study. My name is [researcher name], and I’m a researcher here at the [research institute name] at [hospital name]. In this study, we’re interested in exploring the experiences of working and looking for work among youth with non-visible disabilities, who identify as belonging to a minority race or ethnicity.

Please let me know if you have any questions before we start. Feel free to let me know if you would like to take a break at any time or if there are any questions that you would like to skip or do not feel comfortable with.

**DEMOGRAPHICS**

1. I would first like to ask you some demographic questions

- 1. **Age**___
  2. **Gender:**
- Male
- Female
- Transgender
- Trans male/trans man
- Trans female/trans woman
- Non-binary
- Cisgender
- Intersexual
- Queer
- Questioning
- Two spirited
- I identify as (please describe):___________
  1. * **Ethnic origin refers to a person’s ethnic or cultural origins. Ethnic groups have a common identity, heritage, ancestry, or historical past, often with identifiable cultural, linguistic, and/or religious characteristics. If you are comfortable sharing, can you please tell us what is your ethnic or cultural origin(s**)? For example: Canadian, Chinese, East Indian, English, Italian, Filipino, Scottish, Irish, Portuguese, German, Polish, Dutch, French, Jamaican, Pakistani, Iranian, Sri Lankan, Korean, Ukrainian, Lebanese, Guyanese, Somali, Colombian, Jewish, etc.
  2. ****In our society, people are often described by their race or racial background. These are not based in science, but our race may influence the way we are treated by individuals and institutions. Which category (or categories) best describes you?**
- Black (e.g., African, African Canadian, Afro-Caribbean descent)
- East Asian (e.g., Chinese, Japanese Korean, Taiwanese descent)
- Indigenous (First Nations, Inuk/Inuit, Métis)
- Latin American (e.g., Hispanic or Latin American descent)
- Middle Eastern (e.g., Arab, Persian, West Asian—Afghan, Egyptiona, Iranian, Kurdish, Lebanese, Turkish descent)
- South Asian (e.g., Bangladeshi, Indian, Indo-Caribbean, Pakistani, Sri Lankan descent)
- Another race category
- Prefer not to disclose
  1. **What is your highest level completed of post-secondary education:**
- Currently in high school
- High school diploma
- College, CEGEP, or other non-university certificate or diploma
- University certificate or diploma below bachelor level
- Bachelor’s degree
- Degree in medicine, dentistry, veterinary medicine or optometry
- Master’s degree
- Earned doctorate
- Other
  1. **Please state the disability/condition(s) you have:**

- Brain injury
- Developmental disability (e.g., autism spectrum disorder, intellectual disability)
- Learning disability (e.g., dyslexia, attention deficit)
- Mental illness or psychological disorder (e.g., depression, anxiety, bipolar disorder, schizophrenia)
- Sensory disability (e.g., hearing impairment, sensory processing disorder)
- Other: ______

**MAIN INTERVIEW QUESTIONS**

2. What is your employment status?

- (employed (part / full time) / volunteering (*if yes, probe for the following*):
  - (type and size of company and location)
  - How long have you been working for XX company?
  - How did you find the job?
  - Can you describe what you do on a typical day?
  - What do you like most about working / volunteering here? (probe for inclusion)
  - Do you have any opportunities for professional development or advancement? (probe for type of opportunities; or why lack of opportunities)

1. Have you experienced any challenges in finding employment? (probe for accessibility, accommodations, discrimination, disability disclosure)
   1. In what ways do you feel that your identity (racial, ethnic, disability) has influenced your experiences with looking for work? (probe for experiences, e.g. perceived discrimination during interviews, follow-ups with potential employers, etc.)
   2. What strategies did you use to address these challenges (probe for individual coping mechanisms, peer support, community services/supports)
2. did you disclose your disability to your employer / potential employers?
   1. (*if so, how and when did you do this?)*
      1. why did you decide to disclose?
      2. What accommodations did you ask for?
      3. What response did you receive to your disclosure? How did the response compare to your hopes and expectations?
   2. Have you disclosed your disability to your co-workers?
      1. (if so, why did you decide to disclose, and did anything change after you disclosed?)
   3. *If they have not disclosed:* Can you tell us why you have not disclosed?
      1. Did your racial/ethnic identity play a role in your decision not to disclose? (If so, can you please elaborate?)
3. Do you feel that your racial or ethnic identity influences your employment experiences?
   1. In what ways do you feel that your racial and/or ethnic identity has played a role in shaping your day-to-day experiences at work?
   2. Do you feel included, respected and valued by your employer and co-workers? (if no, probe why not).
   3. In what ways do you feel that your identity has played a role in shaping your employment/career goals?
4. Has your employer/workplace held any training or events related to equity, diversity and/or inclusion?
   1. *If yes:*
      1. Was ableism discussed as part of the training/event(s)?
      2. Was racism discussed?
   2. What was your impression of the training/event(s)? (Do you feel like it was helpful or useful? What were co-workers’ reactions?)
   3. Have these events/training/discussions impacted your decision to disclose/not disclose?
5. What advice do you have for others who have a disability who may be having a difficult time finding employment?
   1. What more could/should be done to encourage workers with non-visible disabilities to disclose their disabilities?
6. Is there anything else that you would like to mention that we did not get a chance to talk about?

*Adapted from Ontario Ministry of Health, “*Collecting information on Ethnic Origin, Race, Income, Household Size, and Language Data: A resource for data collectors*”, <https://www.publichealthontario.ca/-/media/documents/ncov/he/2021/03/aag-race-ethnicity-income-language-data-collection.pdf?la=en>

** Adapted from Canadian Institute for Health Information, *“Guidance on the use of standards for race-based and indigenous identity data collection and health reporting in Canada”,* *[https://www.publichealthontario.ca/-/media/documents/ncov/he/2021/03/aag-race-ethnicity-income-language-data-collection.pdf?la=en](#_Hlk145583932" \s "1,6417,6546,0,,https://www.publichealthontario.)*
